# Learning simple and complex artificial grammars in the presence of a semantic reference field: effects on performance and awareness

**DOI:** 10.3389/fpsyg.2015.00158

**Published:** 2015-02-19

**Authors:** Esther Van den Bos, Fenna H. Poletiek

**Affiliations:** ^1^Institute of Psychology, Leiden UniversityLeiden, Netherlands; ^2^Max Planck Institute for PsycholinguisticsNijmegen, Netherlands

**Keywords:** artificial grammar learning, implicit learning, complexity, reference field, awareness, higher order dependencies, finite state grammars

## Abstract

This study investigated whether the negative effect of complexity on artificial grammar learning could be compensated by adding semantics. Participants were exposed to exemplars from a simple or a complex finite state grammar presented with or without a semantic reference field. As expected, performance on a grammaticality judgment test was higher for the simple grammar than for the complex grammar. For the simple grammar, the results also showed that participants presented with a reference field and instructed to decode the meaning of each exemplar (decoding condition) did better than participants who memorized the exemplars without semantic referents (memorize condition). Contrary to expectations, however, there was no significant difference between the decoding condition and the memorize condition for the complex grammar. These findings indicated that the negative effect of complexity remained, despite the addition of semantics. To clarify *how* the presence of a reference field influenced the learning process, its effects on the acquisition of two types of knowledge (first- and second-order dependencies) and on participants' awareness of their knowledge were examined. The results tentatively suggested that the reference field enhanced the learning of second-order dependencies. In addition, participants in the decoding condition realized when they had knowledge relevant to making a grammaticality judgment, whereas participants in the memorize condition demonstrated some knowledge of which they were unaware. These results are in line with the view that the reference field enhanced structure learning by making certain dependencies more salient. Moreover, our findings stress the influence of complexity on artificial grammar learning.

## Introduction

Early theories of implicit learning (Reber, 1976; Hayes and Broadbent, 1988) proposed that, while simple structures can be learned explicitly, complex structures can best be learned implicitly: by observing exemplars without any intention to learn a structure and without complete awareness of the acquired knowledge. Implicit learning would be suitable for complex regularities, because it would involve unselective storage of the frequency of co-occurrence of all elements present, whereas explicit learning would be limited by the capacity of working memory (Hayes and Broadbent, 1988). Reber (1976) provided some support for this view by demonstrating that participants acquired more knowledge of a finite state grammar by simply memorizing exemplars without knowing that they had been generated according to rules than by trying to figure out the underlying rules.

However, Van den Bos and Poletiek (2008) showed that implicit learning of an artificial grammar, though more effective than explicit learning, was negatively affected by the complexity of the grammar. The higher a grammar's topological entropy (Bollt and Jones, 2000), a measure reflecting the number of unique exemplars of any given length that the grammar can generate, the lower was the participants' performance on a grammaticality judgment test. In particular for items containing multiple violations of second order dependencies, in which the next element in a sequence can be predicted on the basis of two previous elements, performance deteriorated with increasing complexity. This study raised the question of how highly complex structures can be learned. The present study addressed this question by exploring the effect of adding semantic reference. Performance on various types of items and awareness were examined to clarify the workings of a semantic reference field.

The presence of semantic information has been proposed to affect the learning (Moeser and Bregman, 1972; Amato and MacDonald, 2010; Poletiek and Lai, 2012; Poletiek and Monaghan, unpublished manuscript) and processing (Howard and Ballas, 1980; Spivey et al., 2002) of complex structures in natural language. Making exemplars meaningful by adding a reference field (e.g., visual illustrations of scenes they refer to) has often been shown to enhance the learning of phrase structure grammars, which specify the relations between classes of elements (Nagata, 1976; Mori and Moeser, 1983; Meier and Bower, 1986; Valian and Coulson, 1988; Amato and MacDonald, 2010; see also Van den Bos et al., 2012). Moreover, two such studies have shown that the presence of a visual reference field can compensate negative effects of complexity of the grammar (Moeser and Bregman, 1972; Nagata, 1977). Regarding finite state grammars, which consist of rules pertaining to specific elements rather than categories, Howard and Ballas (1980) demonstrated an effect of semantic interpretability of acoustic patterns on structure learning. However, it remains to be established whether a semantic reference field can compensate for complexity of a finite state grammar, when it is learned implicitly (Van den Bos and Poletiek, 2008).

Furthermore, it is unclear *how* reference fields produce their beneficial effects on structure learning. Apart from the possibility that exemplars are easier to process when they are meaningful (Moeser and Bregman, 1972; Amato and MacDonald, 2010), structure learning may also be facilitated by co-presence of the dependent elements in both the exemplar and the reference field (Amato and MacDonald, 2010). Additional correlated cues may help to constrain the learner's hypothesis space (Van den Bos et al., 2012). Finally, some studies using phrase structure grammars proposed that the reference field heightened the salience of phrases constituting the exemplars (Morgan and Newport, 1981; Meier and Bower, 1986; Poletiek and Lai, 2012). Adding a reference field had the same effect as introducing spatial grouping (Morgan and Newport, 1981) and suffix markers (Meier and Bower, 1986).

If a reference field enhances the salience of the structural units, this may have implications for the type of knowledge participants acquire and their awareness. In particular, when aspects of the grammar are made salient, they are likely to capture attention. Attentional processing has been proposed as a necessary requirement for the learning of higher order dependencies, but not first-order dependencies (Keele et al., 2004; Hayes and Lim, 2013). Therefore, higher order dependencies may benefit more than first-order dependencies when the reference field makes both regularities salient. In addition, participants tend to become aware of their knowledge of salient aspects of the structure (Van den Bos and Poletiek, 2010). Thus, in as far as effects of the reference field depend on making aspects of the grammar salient, one might expect that it also increases awareness of these aspects.

To our knowledge, only the study by Amato and MacDonald (2010) addressed the question of whether participants were aware of the knowledge they acquired in the presence of a reference field. Participants learned a miniature artificial language describing monsters acting on objects. Regularities were reflected in the sentences of the artificial language as well as the accompanying pictures of monsters. Because certain combinations of monsters, actions and objects were more frequent than others, the specific combination of first and second words in the sentences (denoting the monster and the action) made one of the possible 5th words (denoting the object) highly probable. Although learning of these dependencies was demonstrated by reduced reading times, sentence and picture completion tasks showed no awareness of the dependencies.

However, these findings cannot readily be generalized to AGL. Dienes and Scott (2005) distinguished between structure knowledge (i.e., knowledge of the structure of training items) and judgment knowledge (i.e., knowledge that a test item does or does not have the same structure as the training items). Reading times are likely to reflect structure knowledge, because participants are not required to make a comparison between training items and test items. In contrast, the grammaticality judgment test used in AGL-experiments involves both structure knowledge and judgment knowledge. Because unconscious structure knowledge is compatible with both conscious and unconscious judgment knowledge (Dienes and Scott, 2005), the effect of a reference field on judgment knowledge cannot be inferred from the findings of Amato and MacDonald (2010).

The present study addressed two main questions. First we investigated whether the negative effect of increasing grammar complexity on learning finite state grammars could be compensated by adding a semantic reference field. The exemplars of two finite state grammars (one simple and one complex) were made meaningful by adding visual illustrations; each exemplar referred to a train with wagons of specific shapes and colors. We expected that the simple grammar would be learned regardless of the presence of a reference field, but that performance on the complex grammar would be enhanced by the reference field. Second, to explore how a reference field may affect the learning process, we investigated whether adding a reference field would differentially affect the acquisition of first- and second-order dependencies and participants' awareness of their knowledge. If the reference field would enhance the acquisition of second-order dependencies in particular and would be associated with increased awareness, this would suggest that the reference field works by increasing the salience of dependencies.

## Method

### Participants

There were 102 participants in this study (18 male, 84 female; 17–37 years of age, *M* = 21.13, *SD* = 3.55). All participants were undergraduate students of Leiden University, who received either course credits or money for their participation. The reward depended on the duration of the experiment. The study was carried out in full accordance with the ethical principles of the Declaration of Helsinki and the regulations of the Department of Psychology.

### Design

The experiment consisted of an induction phase and a test phase. There were three independent variables. Firstly, the task in the induction phase was varied between participants. Thirty-six participants were instructed to memorize the exemplars of an artificial grammar without being presented with a reference field (memorize). Thirty-six other participants were presented with a reference field and received instructions to decode the exemplars and decide whether or not they described one of two trains (decode). To prepare for their task, these participants first learned which object feature each letter was associated with. For the remaining 30 participants there was no induction phase; they formed the control group. Secondly, the complexity of the grammar was varied between participants. One half of the participants with each task worked with a simple grammar; the other half worked with a complex grammar. Finally, different types of exemplars were presented on the grammaticality judgment test (varied within subjects) to address the learning of first and second-order dependencies. The dependent variable was the proportion of correct grammaticality judgments in the test phase.

### Materials

**Figure 1:**
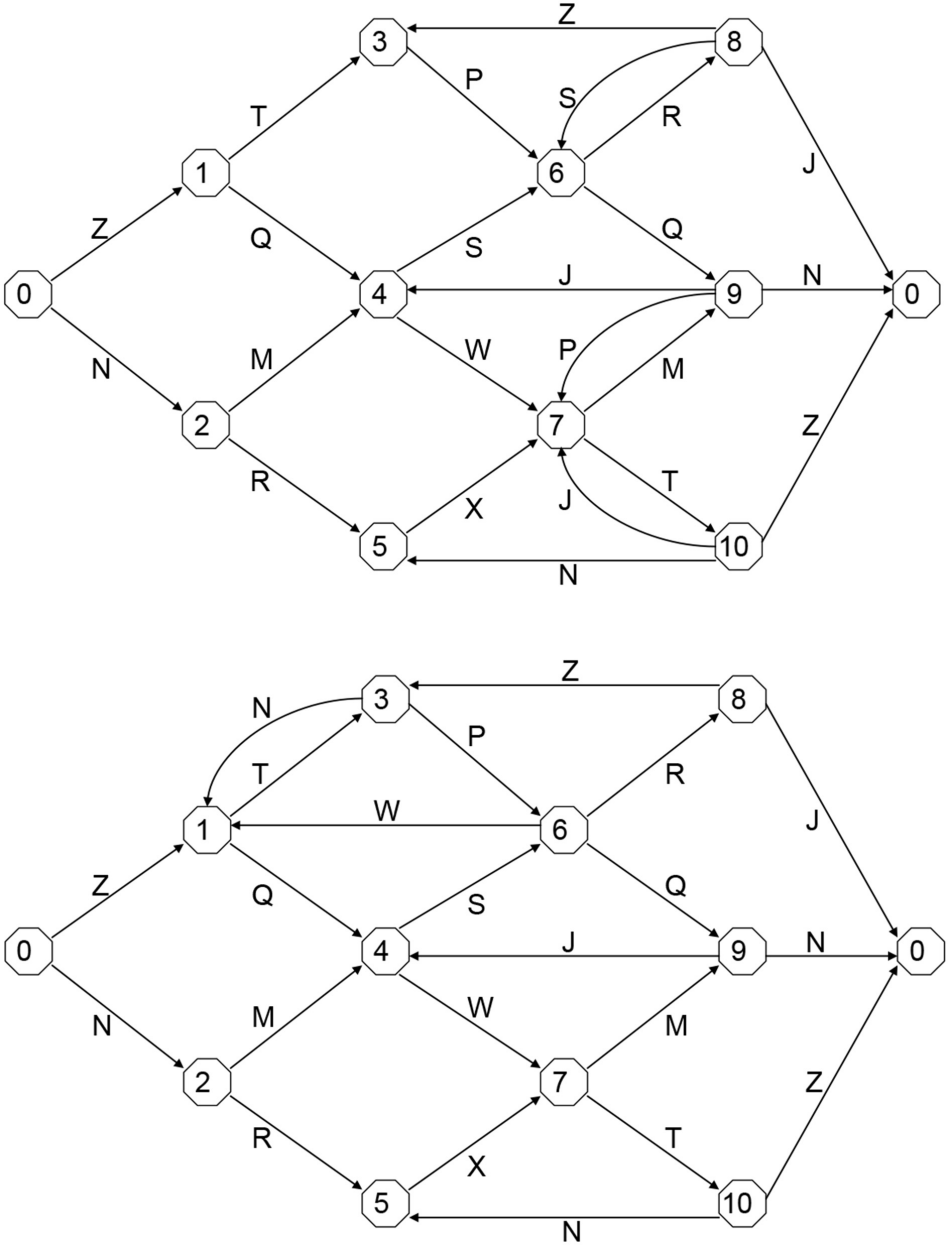
**The simple (top) and complex (bottom) finite state grammars used in the present study**.

The stimuli were exemplars of two of the finite-state grammars used to vary complexity by Van den Bos and Poletiek (2008). Both grammars consisted of the same 11 states and used the letters J, M, N, P, Q, R, S, T, W, X, and Z. Complexity was manipulated by creating different connections between the states (see Figure 1). Topological entropy (Bollt and Jones, 2000) was 0.71 for the simple and 2.05 for the complex grammar.

A computer program generated a set of 120 unique exemplars of 5–11 letters for each grammar. Of each set, 60 exemplars were assigned to the induction phase and 48 to the test phase, so that the paths of the grammar were represented according to the same ratio in both phases of the experiment (see Table A1). Five of the remaining exemplars were used on practice trials in the test phase; the rest was discarded. In the decoding condition, the same exemplars were used on the practice trials of the induction phase, while number strings unrelated to the grammars were used in the memorize condition. On the experimental trials of the induction phase, the exemplars were the same for both tasks. The exemplars presented in the test phase were the same for all conditions using each grammar.

In the decoding condition, each letter referred to either a shape (M = circle, Q = diamond, R = triangle, T = rectangle) or a color (J = light green, N = yellow, P = dark green, S = purple, W = red, X = blue, Z = orange). To learn these associations, the letters were first presented together with pictures of shapes and color patches. In the subsequent induction phase, each exemplar was accompanied by two pictures of trains pulling wagons with various shapes of various colors. One third of the exemplars referred to the train in the upper picture, one third referred to the train in the lower picture and one third referred to a train that was not presented.

Two rules of reference (of which participants were not informed) were applied to create the correct train for each exemplar. First, if a color was followed by a shape, it applied to that shape. Second, if a color was followed by another color, it produced a stripe on the preceding shape. For example, NRXTZ referred to a train pulling a wagon with a yellow triangle and a wagon with a blue rectangle with an orange stripe. Incorrect alternatives were created by substituting a random color or shape for one specified by the exemplar. Substitutions were balanced over the position and frequency of the substituted letter.

As in Van den Bos and Poletiek (2008), four types of exemplars were used on the grammaticality judgment test: 1 grammatical and 3 ungrammatical. The ungrammatical exemplars were created by switching two adjacent inner letters (excluding the first and last). Switching letters could result in violations of first-order dependencies (an illegal sequence of two letters) and violations of second-order dependencies (a sequence of two letters that is illegal given the letter preceding it). Switching two inner letters of a string always affects three transitions, thus producing one of four theoretically possible combinations of violations: 3 first-order violations, 2 first- and 1 second-order violations, 1 first- and 2 second-order violations, or 3 second-order violations. Because switching two letters rarely resulted in 3 second-order violations with the current grammars, this type of ungrammatical item was not presented. The three other types were represented equally among the 24 ungrammatical items. For each grammar, there were 8 items containing 3 violations of first-order dependencies, 8 items containing 2 first-order and 1 second-order violation and 8 exemplars containing 1 first-order and 2 second-order violations. The remaining 24 exemplars were unaltered grammatical strings.

### Procedure

Participants were tested individually in a dimly lit test booth. They were seated in front of a computer monitor, on which the stimuli were displayed, at a distance of about 50 cm. They reacted by pressing keys on a keyboard. The procedure for the induction phase depended on the participants' task.

Participants in the memorize condition were instructed to study each exemplar for a memory test. They were presented with 5 practice trials, after which they were notified that the real task began. The 60 experimental trials were presented in random order. Each trial began with a fixation cross in the middle of the screen. After 1 s the cross was replaced by a grammatical exemplar centered at the fixation point. The exemplar was displayed for 5 s. Presentation time was held constant across conditions and based on pilot sessions with the decoding task. When the exemplar had disappeared, participants were prompted to reproduce it. After pressing the enter-key, participants were again presented with the original exemplar for 2 s so that they could check their answer. Finally, the screen turned blank for 1 s before the next trial began.

The decoding condition started with a vocabulary acquisition phase. Participants were informed that this was a necessary preparation for their next task. A screen pairing each letter with its corresponding color or shape was presented and participants were instructed to study it until they knew the referent of each letter. After pressing the enter-key, they received a vocabulary test. The letters were presented in random order together with three numbered pictures of colors or shapes. Participants had to indicate which picture each letter referred to by pressing 1, 2 or 3 and received feedback after each trial. This study-test procedure was repeated three times.

In the subsequent induction phase, participants were informed that they would see two pictures of trains and one letter string. They were instructed to press 1 if the string referred to the upper train, 2 if it referred to the lower train or 3 if it referred to neither. Participants received no prior information on how the shapes and colors denoted by each letter had to be combined. Each trial (5 practice and 60 experimental) started with the presentation of the trial number. After 1 s, an exemplar of the grammar was presented in the lower half of the screen together with two pictures of trains presented above each other in the upper half. When the participant pressed 1, 2 or 3, the two pictures were replaced by one picture of the correct referent, accompanied by the word “Correct!” if the answer was right. After 5 s, the feedback screen was followed by the next trial.

The induction phase was followed by the grammaticality judgment test. Experimental participants were informed that the previously presented exemplars had been generated according to a complex set of rules. They were instructed to judge whether or not new exemplars followed the same rules. Control participants received a similar instruction, which did not refer to the induction phase. Participants were instructed to press the “j”-key (for “ja”: “yes”) if they thought that an exemplar followed the rules or the “n”-key (for “nee”: “no”) if they thought that it did not follow the rules. In addition, they were required to indicate their confidence in each judgment on a scale from 1 (very little) to 5 (very much) by pressing one of the number keys on the keyboard.

Participants received 5 practice trials followed by 48 experimental trials, presented in random order. Each trial began with a fixation cross appearing in the middle of the screen. After 1 s the cross was replaced by an exemplar centered at the fixation point. When the participant pressed the “j” or “n”-key, the screen turned blank for 1 s. Subsequently, the confidence scale was presented until the participant pressed a number from 1 to 5. A final blank screen separated two consecutive trials by 1 s.

After the test, participants were thanked for their participation. The duration of the experiment varied from 10 min for the control condition to 30 min for the memorize condition and 45 min for the decoding condition.

### Analyses

The data from the grammaticality judgment test were analyzed by means of a 2 × 3 × 4 mixed-model analysis of variance (ANOVA) with grammar (simple vs. complex) and task in the induction phase (memorize vs. decode vs. control) as between-subjects variables and type of exemplar (grammatical vs. 3 first-order violations vs. 2 first, 1 second-order violation vs. 1 first, 2 second-order violations) as within-subjects factor.

Confidence ratings were used to assess participants' awareness of their knowledge. A study comparing different subjective measures in the context of artificial grammar learning showed that confidence ratings captured the largest range of consciousness (Wierzchon et al., 2012). Specifically, the guessing criterion and the zero-correlation criterion proposed by Dienes et al. (1995) were used to assess participants' awareness of their judgment knowledge (Dienes and Scott, 2005). When participants perform above chance while having so little confidence in their judgments that they claim to be guessing, they possess knowledge of which they are unaware in the sense that they lack meta-knowledge (i.e., unconscious judgment knowledge). The guessing criterion provides a meaningful distinction, as it has been shown to distinguish between knowledge that can be retrieved under divided attention and knowledge that cannot. The proportion of correct grammaticality judgments was computed for trials on which participants had (very) little confidence (ratings 1 and 2). This was used as the dependent variable in a 2 × 3 ANOVA with grammar and task in the induction phase as between-subjects variables. According to the zero-correlation criterion, participants are unaware of their knowledge when confidence ratings are unrelated to judgment accuracy. Conversely, conscious judgment knowledge is indicated by higher confidence ratings for accurate judgments than for inaccurate judgments. Participants' mean confidence ratings for incorrect judgments were subtracted from their mean confidence ratings for correct judgments (Dienes et al., 1995). This difference score was used in a 2 × 3 ANOVA with grammar and task in the induction phase as between-subjects variables.

## Results

The ANOVA on the proportion of correct grammaticality judgments showed a main effect of grammar [*F*_(1, 96)_ = 12.317, *MSE* = 0.045, *p* = 0.001]: performance was better with the simple grammar (*M* = 0.629) than with the complex grammar (*M* = 0.555). In addition, there were significant effects of task in the induction phase [*F*_(2, 96)_ = 30.864, *MSE* = 0.045, *p* < 0.001] and type of exemplar [*Wilks λ* = 0.698, *F*_(3, 94)_ = 13.558, *p* < 0.001]. The main effects were modified by significant interactions between grammar and task in the induction phase [*F*_(2, 96)_ = 3.372, *p* = 0.038] and between task in the induction phase and type of exemplar [*Wilks λ* = 0.657, *F*_(6, 188)_ = 7.323, *p* < 0.001], which will be examined below.

**Figure 2:**
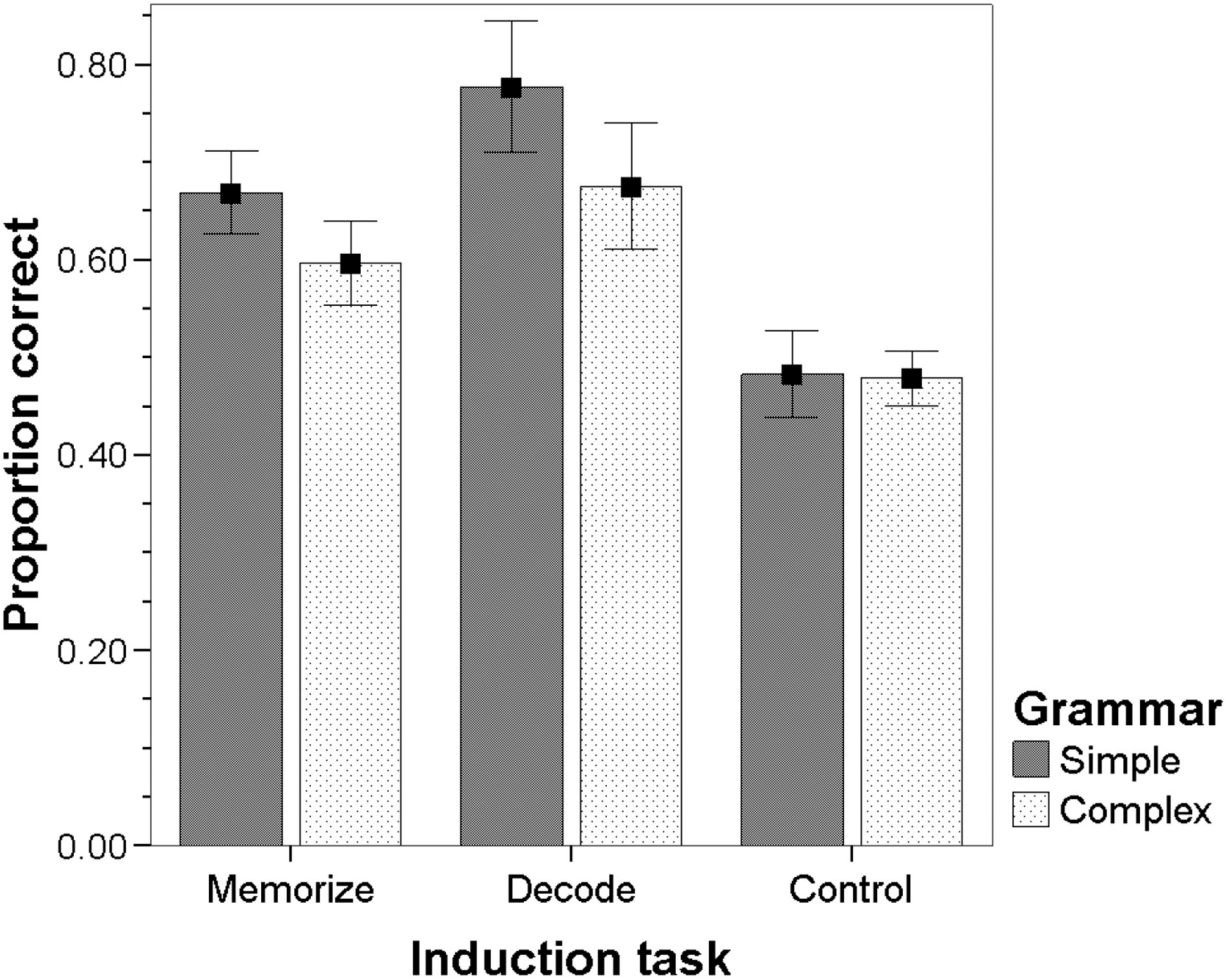
**Mean proportion correct with 95% confidence interval for each task in the induction phase and grammar**.

The interaction between grammar and task in the induction phase is illustrated by Figure 2. For both the simple and the complex grammar, separate ANOVA's showed that the proportion of correct grammaticality judgments over all items depended on the task in the induction phase [simple: *F*_(2, 48)_ = 32.961, *p* < 0.001; complex: *F*_(2, 48)_ = 16.931, *p* < 0.001]. The effects of task in the induction phase were examined using *post-hoc* tests with bonferroni correction. For the simple grammar, the proportion correct was higher in the experimental conditions than in the control condition (*p* < 0.001 for both comparisons) and higher in the decoding condition than in the memorize condition (*p* = 0.009). For the complex grammar, the proportion correct was also higher in the experimental conditions than in the control condition (Memorize: *p* = 0.003; Decode: *p* < 0.001). However, performance in the decoding condition was only marginally significantly higher than in the memorize condition (*p* = 0.057).

The interaction between task in the induction phase and type of test exemplar was examined by performing separate ANOVA's for each type of exemplar. Proportion correct (see Table 1) varied with the task in the induction phase for grammatical exemplars [*F*_(2, 99)_ = 46.832, *p* < 0.001], exemplars with 3 first-order violations [*F*_(2, 99)_ = 8.648, *p* < 0.001] and exemplars with 2 first and 1 second-order violation [*F*_(2, 99)_ = 24.453, *p* < 0.001]. The effects of task in the induction phase were examined using *post-hoc* tests with bonferroni correction. For grammatical exemplars, the proportion correct was higher in the experimental conditions than in the control condition (*p* < 0.001 for both comparisons) and higher in the decoding condition than in the memorize condition (*p* < 0.001). For exemplars with 3 first-order violations, the proportion correct was also higher in the experimental conditions than in the control condition (*p* = 0.001 for both comparisons), but there was no difference between memorize and decoding conditions. For exemplars with 2 first and 1 second-order violation, the proportion correct was again higher in the experimental conditions than in the control condition (Memorize: *p* = 0.002, Decode: *p* < 0.001) and higher in the decoding condition than in the memorize condition (*p* = 0.001). In contrast, there was no difference between the tasks in the induction phase for exemplars with 1 first and 2 second-order violations [*F*_(2, 99)_ < 1]. Neither memorizing nor decoding enabled participants to perform better than the control group on this type of exemplar.

**Table 1:** **Mean proportion of correct grammaticality judgments with standard deviations for each type of exemplar and induction task**.

**Type of exemplar**	**Task**		
	**Memorize**	**Decode**	**Control**
Grammatical	0.667 (0.119)	0.821 (0.173)	0.487 (0.115)
3 first-order violations	0.680 (0.178)	0.676 (0.220)	0.505 (0.166)
2 first, 1 second-order violation	0.590 (0.184)	0.759 (0.221)	0.415 (0.189)
1 first, 2 second-order violations	0.534 (0.195)	0.467 (0.201)	0.503 (0.228)

*Standard deviations are in parentheses*.

### Awareness

The ANOVA on the proportion of correct low-confidence responses only showed a main effect of task in the induction phase [*F*_(2, 81)_ = 3.417, *MSE* = 0.044, *p* = 0.038]. *Post-hoc* tests with bonferroni correction showed that the proportion correct in the memorize condition (*M* = 0.624, *SD* = 0.156) was marginally significantly higher than in the control condition (*M* = 0.499, *SD* = 0.125; *p* = 0.074), whereas the proportion correct in the decoding condition (*M* = 0.507, *SD* = 0.316) did not differ from the control condition. Thus, participants in the memorize condition showed some knowledge of the grammar while thinking they were guessing, indicating that they possessed some knowledge of which they were unaware. The pattern of results did not change when the number of low-confidence responses was included as a covariate in the analysis; the covariate was not significant [*F*_(1, 80)_ < 1] and the main effect of task in the induction phase remained [*F*_(2, 80)_ = 3.317, *p* = 0.041].

The ANOVA on the difference score of confidence ratings for correct and incorrect judgments also showed only a main effect of task in the induction phase [*F*_(2, 96)_ = 5.822, *MSE* = 0.0.93, *p* = 0.004]. *Post-hoc* tests with bonferroni correction indicated that the difference in confidence between correct and incorrect judgments was larger in the decoding condition (*M* = 0.27, *SD* = 0.31) than in the control condition (*M* = 0.01, *SD* = 0.26, *p* = 0.003), whereas there was no difference between the control condition and the memorize condition (*M* = 0.13, *SD* = 0.33). In sum, compared to the control condition, participants in the decoding condition showed more conscious judgment knowledge, but not more unconscious judgment knowledge. Participants in the memorize condition, in contrast, showed marginally more unconscious judgment knowledge than control participants, whereas they did not show more conscious judgment knowledge.

## Discussion

The present study replicated the negative influence of grammar complexity on implicit learning that was observed by Van den Bos and Poletiek (2008). Participants who worked with exemplars from a simple grammar were better at judging the grammaticality of new exemplars than participants who worked with exemplars from a complex grammar. The main question in the present study was whether the negative effect of complexity could be compensated by adding a reference field. Participants who had to decode the meaning of the exemplars performed better on the grammaticality judgment test than participants who memorized the exemplars, but contrary to expectations, the difference was only significant for the simple grammar. So, the presence of the reference field did not compensate the negative effect of complexity.

The present study also attempted to clarify the workings of the reference field by exploring its effects on the acquisition of first- and second-order dependencies and on participants awareness of their knowledge. Supplying a reference field differentially affected performance on the four types of items presented at test (grammatical, 3 first-order violations, 2 first and 1 second-order violations, 1 first and 2 second-order violations), providing some evidence for the suggestion that the learning of second-order dependencies in particular would benefit from the presence of a reference field. While there was no difference between memorize and decoding conditions on items containing only first-order violations, decoding led to better performance than memorizing on items containing 2 first-order and 1 second-order violations.

However, the finding that performance on items with 1 first and 2 second-order violations was at chance in all conditions suggests that the learning of second-order dependencies was limited. Van den Bos and Poletiek (2008) found that performance on this type of item deteriorated with increasing complexity of the to-be-learned grammar. The present finding suggests that successful performance on those items may have been restricted to the very simplest grammar in that study. The present study did not provide any indication that second-order dependencies were learned in the memorize condition. Participants may have achieved above chance performance using knowledge of first-order dependencies or other characteristics of the stimuli, such as similarity to individual training strings and patterns of letter repetitions (Lotz and Kinder, 2006).

Regarding the question of whether the presence of a reference field would affect participants' awareness of the knowledge they acquired, judgment knowledge was mainly conscious in the decoding condition and mainly unconscious in the memorize condition. In the decoding condition, participants seemed to know whether or not they had relevant knowledge to make a grammaticality judgment. If they had little confidence in their judgment, they were not more likely to be correct than control participants who had not been previously exposed to grammatical exemplars, indicating no unconscious judgment knowledge (Dienes and Scott, 2005). Moreover, higher confidence ratings were associated with accurate judgments on the grammaticality test, indicating conscious judgment knowledge (Dienes and Scott, 2005). Participants in the memorize condition, in contrast, acquired some knowledge of which they were unaware. They did somewhat better than control participants, even when they had little confidence in their judgments, suggesting unconscious judgment knowledge (Dienes and Scott, 2005). There was no evidence for conscious judgment knowledge, because the relation between confidence and accuracy was no stronger in the memorize group (as a whole) than in the control group. However, judgment knowledge may have been conscious in some individual participants.

The finding that participants who were presented with a reference field were, overall, more aware of the knowledge they acquired is in line with the view that reference fields enhance artificial grammar learning by making certain aspects of the structure salient. Identifying a wagon in the reference field always required combining the meanings of two or three individual letters. Consistently processing the exemplars as concatenations of two and three letter chunks is likely to enhance the salience of those chunks and may have led to knowledge of bigrams and trigrams of which participants are aware.

In summary, the results tentatively suggest that the presence of a reference field did not affect the learning of first-order dependencies, but enhanced the learning of second-order dependencies. At the same time, the reference field made participants more aware of the knowledge they acquired. These findings are in line with the view that the learning of higher order dependencies requires attention, while the learning of first-order dependencies may not (Keele et al., 2004; Hayes and Lim, 2013). However, further research involving measures of attention is needed to corroborate this account.

In addition, further research might clarify whether the negative effect of complexity on implicit learning of finite state grammars can be canceled out by other means. Previous studies have demonstrated that artificial grammar learning can be enhanced by optimizing the ordering (Poletiek, 2011) and representativeness (Poletiek and Van Schijndel, 2009; Poletiek and Lai, 2012) of the exemplars presented in the induction phase. However, some facilitating conditions might work for hierarchical grammars, but not finite state grammars: the model by Poletiek (2011) explains that staging the input, which enhances the learning of center-embedded (phrase structure) grammars, does not affect the learning of non-hierarchical (finite state) grammars. The present finding that semantics do not fully compensate for complexity in finite state grammars lines up with this possibility. Likewise, Poletiek and Lai (2012) argue that semantic biases are helpful for learning hierarchical center-embedded grammars in particular.

Studies using reaction time (Remillard, 2008) and reading time measures (Amato and MacDonald, 2010) have demonstrated learning of higher order dependencies in probabilistic structures, which characterize complex finite state grammars. However, the same studies also showed that participants did not recognize the dependencies explicitly. This may suggest that the knowledge one typically acquires of these structures (presumably an unconscious form of structure knowledge) is not represented in a way suitable for making judgments; the representations may not allow the comparison between training and test items, which according to Dienes and Scott (2005) is involved in judgment knowledge.

The present study produced three findings. Firstly, it replicated the finding that implicit artificial grammar learning is hampered by increasing complexity of the grammar. Secondly, it provided further evidence that structure learning can be enhanced by providing a semantic reference field. Participants who decoded the meaning of exemplars from an artificial grammar acquired more knowledge (possibly of second-order dependencies) and became more aware of their knowledge than participants who memorized the exemplars, suggesting that the reference field produced its effect by making certain bigrams and trigrams more salient. Thirdly, the presence of a reference field did not compensate the negative effect of complexity of the grammar on performance on the grammaticality judgment task. Grammars are more easily learned when they represent meaning directly, but semantics may fall short for complex grammatical rules that do not represent meaning in a straightforward way.

### Conflict of interest statement

The authors declare that the research was conducted in the absence of any commercial or financial relationships that could be construed as a potential conflict of interest.
