# Supplementary material for: Learning simple and complex artificial grammars in the presence of a semantic reference field: effects on performance and awareness
Source: Front Psychol. 2015 Feb 19;6:158. doi: 10.3389/fpsyg.2015.00158 (PMC4333800; doi:10.3389/fpsyg.2015.00158)
Supplement: Supplementary file 1 [file DataSheet1.PDF]

Appendix

Table A1. Exemplars Presented in the Induction Phase and the Test Phase for the Simple Grammar and the Complex Grammar.

Simple Grammar		Complex Grammar	
**Induction phase**	**Test phase**	**Induction phase**	**Test phase**
NMSQJSQN	**Grammatical**	NMSQJSQN	**Grammatical**
NMSQJWMN	NMSQN	NMSQJWMN	NMSQN
NMSQPMPMN	NMSRJ	NMSRZNQWMN	NMSRJ
NMSQPTZ	ZTPQN	NMSRZPRJ	ZTPQN
NMSRSQN	NMSRSRJ	NMSWQSQN	ZTNTPRJ
NMSRZPRJ	NMWTJTZ	NMSWTPRJ	NMSQJSRJ
NMWMJSRJ	NRXTJTZ	NMWMJSRJ	NMSRZPQN
NMWMJWMN	ZQSRSRJ	NMWMJWMN	NMWMJSQN
NMWMN	ZTPQPTZ	NMWMJWTNXTZ	NMWMJWTZ
NMWMPMN	NMSQJSRJ	NMWMN	NRXMJWTZ
NMWMPMPMN	NMWMJWTZ	NMWTNXMJWMN	ZQSQJSRJ
NMWMPTJTZ	NRXMJWTZ	NMWTNXTNXTZ	ZQSQJWMN
NMWTJMN	ZQSQJSRJ	NMWTNXTZ	ZQSWQWMN
NMWTNXTZ	ZQWMJWTZ	NRXMJSRJ	ZQWTNXTZ
NRXMJSRJ	ZQWTNXTZ	NRXMJWMJWMN	ZTPQJWMN
NRXMJWMN	NMSQPMPTZ	NRXMJWMN	ZTNTNQWMN
NRXMN	NRXMPMPMN	NRXMJWTNXTZ	ZTNTNTPQN
NRXMPMPTZ	ZQSQPMPMN	NRXMN	ZTNQWMJWTZ

Simple Grammar		Complex Grammar	
**Induction phase**	**Test phase**	**Induction phase**	**Test phase**
NRXMPTJTZ	ZTPQPTJMN	NRXTNXMJSRJ	ZTNTPQJWMN
NRXMPTNXTZ	ZTPRSRSQN	NRXTNXMJWMN	ZTPWTNQWTZ
NRXMPTZ	NRXTJTNXTZ	NRXTNXTNXTZ	NMWTNXMJWTZ
NRXTJMN	NRXTNXTJMN	NRXTNXTZ	NRXTNXMJWTZ
NRXTJMPMN	ZTPQPTNXMN	NRXTZ	NRXTNXTNXMN
NRXTJTJMN	ZTPRSRZPRJ	ZQSQJSQN	ZQWMJWTNXMN
NRXTJTJTZ	NRXTNXTNXMN	ZQSQJWTZ	ZQWTNXTNXTZ
NRXTJTNXMN	**Ungrammatical**	ZQSQN	**Ungrammatical**
NRXTNXMPMN	NWMTZ	ZQSRZNQWTZ	ZTNQRSJ
NRXTNXTJTZ	ZQMWN	ZQSRZPQN	NSMQJWTZ
NRXTNXTNXTZ	NMQSPMN	ZQSWQSQN	NMSTWPQN
NRXTNXTZ	ZQSPQTZ	ZQSWQSRJ	NMTWNXMN
NRXTZ	ZWQMPMN	ZQSWTPRJ	ZQSRZRPJ
ZQSQJSQN	ZQWTMJN	ZQWMJSQN	ZQWMJTWZ
ZQSQJWTZ	NMSQWJTZ	ZQWMJSRJ	ZTNPTWQWTZ
ZQSQN	NMWJMSQN	ZQWMJWMN	NRMXJWMJWTZ
ZQSQPMN	NMTWNXMN	ZQWMJWTNXTZ	NWMTZ
ZQSQPMPTZ	NRXJMSQN	ZQWTNXMJWMN	ZQMWN
ZQSRSQN	ZQSQJMWN	ZQWTNXMN	ZTPJQWTZ
ZQSRZPQN	ZQSRZRPJ	ZQWTNXTNXMN	ZTNTQNSRJ
ZQWMJSQN	ZPTQJSRJ	ZQWTZ	NMWMJWTNMXN

Simple Grammar		Complex Grammar	
**Induction phase**	**Test phase**	**Induction phase**	**Test phase**
ZQWMJSRJ	ZTPQWJMN	ZTNQSQJWTZ	NMWTNXTXNMN
ZQWMJWMN	NMWPMTJMN	ZTNQSQN	NRXTNXJMSQN
ZQWMPTZ	NRMXPTJMN	ZTNQSWQWTZ	ZQWTNMXJWTZ
ZQWTJMPMN	NRTXJMPTZ	ZTNQWMJWMN	ZNTQWMN
ZQWTJTZ	ZQSQTPJTZ	ZTNQWTNXTZ	NMSQWSRJ
ZQWTNXMN	ZWQTJMPTZ	ZTNQWTZ	NMWSQWMN
ZQWTZ	ZPTRSQPMN	ZTNTNQSQN	ZQWSQWTZ
ZTPQJSQN	NXRMPTNXMN	ZTNTNQWTZ	ZSQWTPQN
ZTPQJWTZ	NRXNTXMPTZ	ZTNTNTPRJ	ZTPQWSRJ
ZTPQPMN	ZTPRPZRSQN	ZTNTPQJWTZ	ZQSRZQNWMN
ZTPQPMPMN	ZTPRZPRZQPN	ZTNTPQN	ZTNSQWQWMN
ZTPQPTJTZ		ZTNTPWQWMN	
ZTPQPTNXTZ		ZTPQJSQN	
ZTPRJ		ZTPQJSRJ	
ZTPRSQPTZ		ZTPRJ	
ZTPRSRJ		ZTPRZPQN	
ZTPRSRSRJ		ZTPWQSQN	
ZTPRSRZPQN		ZTPWQWTZ	
ZTPRZPQPMN		ZTPWTNQWMN	
ZTPRZPRSRJ		ZTPWTPQN	
ZTPRZPRZPRJ		ZTPWTPRJ	

Note. Violations are underlined.
